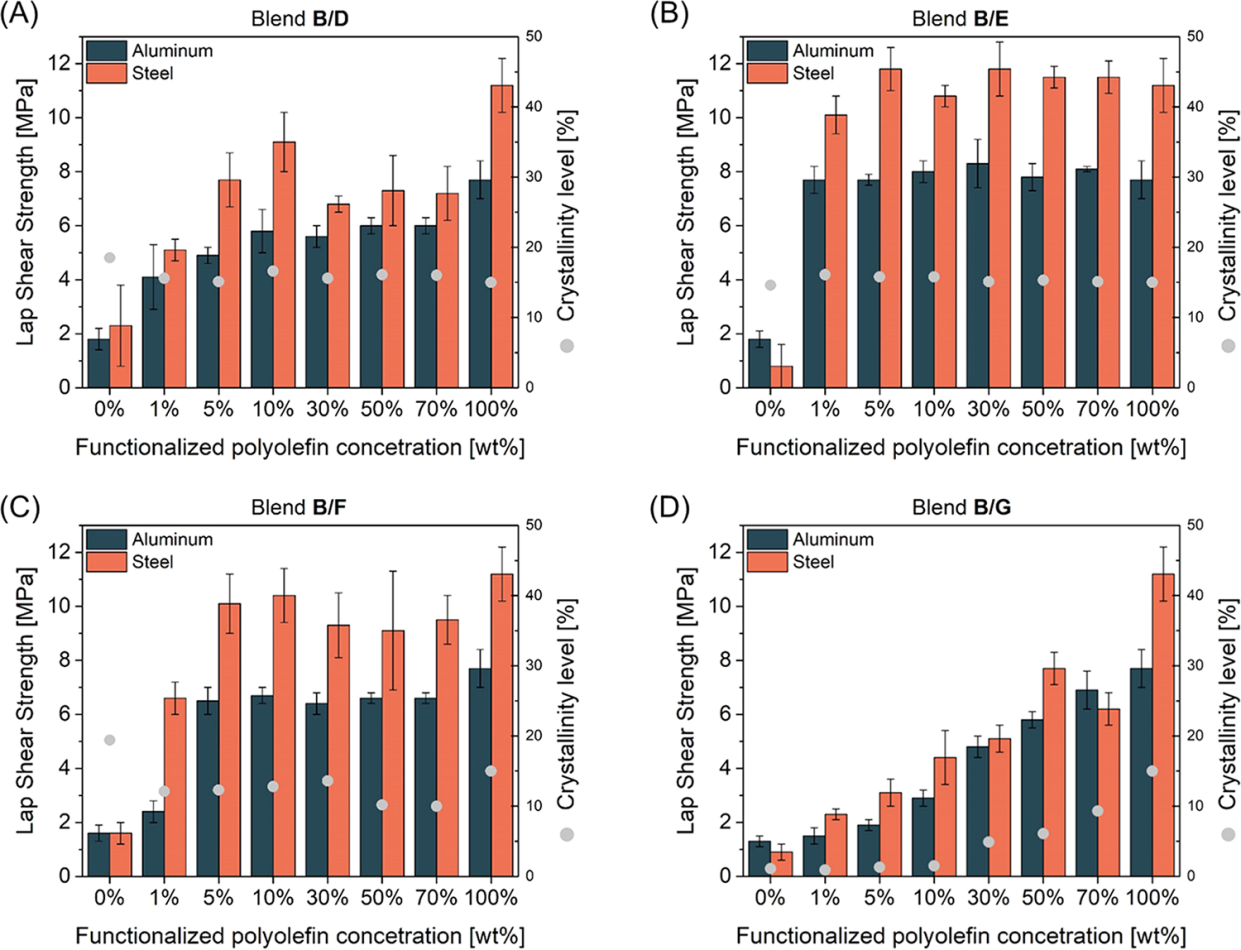# Correction to “Tuning the Adhesive Strength of Functionalized Polyolefin-Based Hot Melt Adhesives: Unexpected Results Leading to New Opportunities”

**DOI:** 10.1021/acs.macromol.5c01967

**Published:** 2025-08-05

**Authors:** Jakub Kruszynski, Weronika Nowicka, Farhan Ahmad Pasha, Lanti Yang, Artur Rozanski, Miloud Bouyahyi, Ralf Kleppinger, Lidia Jasinska-Walc, Rob Duchateau

Figure 7D in the published paper is the same as Figure 7A. Now, the corrected Figure 7D represents correct data for blends B/G: